# Microbial transformation of sewage sludge to biolipid-based fuel using potential oleaginous bacteria *Streptomyces* sp.

**DOI:** 10.3389/fmicb.2025.1551264

**Published:** 2025-05-14

**Authors:** Sana Akbar, Muzammil Anjum, Samia Qadeer, Rab Nawaz, Zepeng Rao, Habib Ullah, Abdulaziz Alamri, Mohamed A. El-Tayeb

**Affiliations:** ^1^Department of Environmental Sciences, Institute of Soil and Environmental Sciences, Pir Mehr Ali Shah Arid Agriculture University, Rawalpindi, Pakistan; ^2^Department of Environmental Sciences, Allama Iqbal Open University, Islamabad, Pakistan; ^3^Department of Earth Sciences and Environment, Faculty of Science and Technology, University Kebangsaan Malaysia (UKM), Bangi, Malaysia; ^4^Innovation Center of Yangtze River Delta, Zhejiang University, Zhejiang, China; ^5^Department of Environmental Science, Zhejiang University, Hangzhou, China; ^6^Department of Biochemistry, College of Science, King Saud University, Riyadh, Saudi Arabia; ^7^Department of Botany and Microbiology, College of Science, King Saud University, Riyadh, Saudi Arabia

**Keywords:** biodegradation, oleaginous bacteria, *Streptomyces* sp., sludge, transesterification, biolipid, biofuel

## Abstract

**Introduction:**

The high proportion of sludge generation worldwide has sparked interest in utilizing it for alternative purposes. Among different potential applications, using sludge as a substrate for oleaginous bacteria is a relatively novel approach. The study was conducted to harness *Streptomyces* sp. to produce bio-lipids and their further processing for biofuel through transesterification.

**Methods:**

Sewage sludge was obtained from the I-9 treatment plant, Islamabad; after initial characterization the unprocessed sludge was optimized viz.: incubation time (24–96 h), inoculation rate (5–15%), pH levels (4–9), temperature (25–40°C), agitation (0–250 RPM), nitrogen sources (yeast, urea, ammonium chloride, and ammonium nitrate), and carbon sources (glucose, sucrose, starch, and dextrose). The qualitative analysis of the stored bio-lipids was performed using Fourier-transform infrared spectroscopy (FTIR) and Gas Chromatography Mass Spectrometry (GC–MS).

**Results and discussion:**

The maximum reactor performance was achieved with 40% lipid accumulation (gravimetric basis) in the dry cell biomass of *Streptomyces* sp. The results indicated the presence of C-H (Alkane), with additional phenolic and alcoholic bonds through FTIR, whereas the GC–MS results indicated the presence of palmitic acid and oleic acid as the most recurring compounds. This highlights the strong potential of *Streptomyces* sp. for biolipid based fuel production using sludge as a substrate. The contents of the extract (i.e., bio-lipids) were successfully transesterified to produce biofuels from the stored lipids. The findings indicated that the use of *Streptomyces* sp. potentially provides a dual benefit of reducing organic loading from the sludge along with biofuel production under optimized reactor conditions.

## Graphical abstract

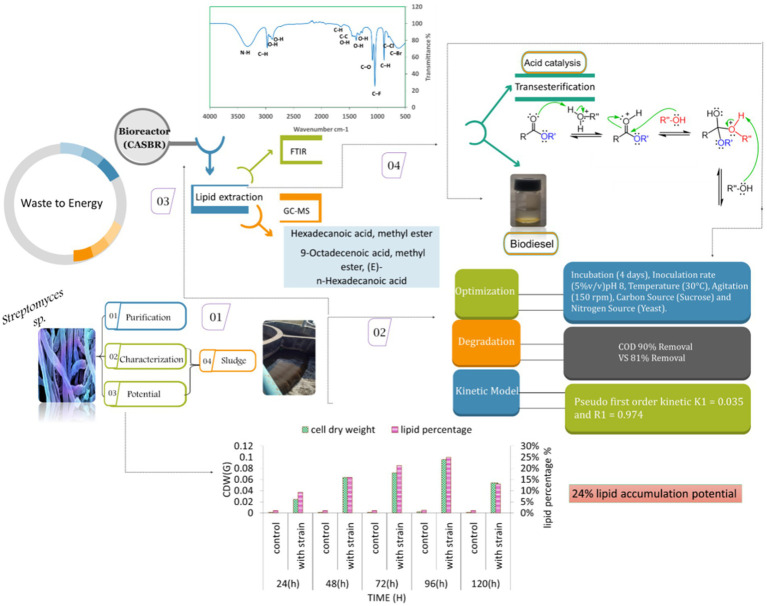

## 1 Introduction

As fossil fuel sources diminish, the importance of renewable fuels for future generations becomes increasingly evident. According to York and Bell (2019) approximately 84% of the world’s current energy consumption is fueled by fossil fuels, contributing to heightened greenhouse gas emissions. Renewable biofuels have garnered significant interest in solving these environmental challenges (Cea et al., 2015). Oleaginous species have emerged as significant contributors to bio-lipid-based fuel manufacturing among diverse microorganisms, including fungi, bacteria, and microalgae (Chintagunta et al., 2021). These microorganisms can accumulate lipids within their cells, especially Triacylglycerol (TAG), making them a desirable source for biofuel production (Chew et al., 2018). According to Abdellatief et al. (2023), global liquid fuel consumption is expected to reach 122 million barrels per day by 2040, up from 87 million barrels per day in 2010. This continual increase in fuel usage poses problems as it depletes fossil fuel supplies and exacerbates environmental issues. To address these concerns, there is an escalating need to investigate alternative fuels (Ambaye et al., 2021). In recent years, there has been a growing interest in using bacteria as a viable source of oil for biofuel generation (Alalwan et al., 2019). Biodiesel, offering a renewable and cleaner fuel for engines, has demonstrated potential as a response to today’s environmental challenges (Manaf et al., 2019) The utilization of oil-producing bacteria, often referred to as single-cell oils, has garnered global attention due to their ability to accumulate oil and their economic viability for biofuel production (Patel et al., 2020).

A crucial step toward environmentally friendly and sustainable biofuel production is the investigation of alternative feedstocks, such as microbial or non-edible vegetable oils, for biodiesel synthesis (Athar and Zaidi, 2020). The choice of substrate is pivotal in biofuel production, and various types of waste, including food waste and sewage sludge, have been explored as potential substrates for lipid accumulation (Smoliński et al., 2019). Due to the rapid growth of the global economy, the volume of wastewater treated in urban and rural wastewater treatment plants has increased day by day (Deng et al., 2025; Xu et al., 2025). Wastewater treatment processes generate substantial amounts of sludge, which contains a high concentration of TAG, making it attractive for biofuel production (Suresh et al., 2021). Primary sludge obtained during the initial treatment stage typically exhibits a higher lipid content than secondary sludge, which consists of microbial cells released after the disruption of cell structures (Bora et al., 2020). TAG production has been extensively present in actinomycetales order filamentous bacterial species, including *Mycobacterium, Streptomyces, Nocardia, and Rhodococcus* (Mothana et al., 2022). *Streptomyces* sp. belongs to antibiotics (Koreti et al., 2022), due to its capacity to accumulate lipids, constituting more than 20% of its biomass, this bacterium is categorized as oleaginous biomass (Sunish and Thazeem, 2023).

It is well-recognized that the availability of carbon and nitrogen sources, aeration, and temperature play a pivotal role in the degradation of waste and in modulating the concentration and composition of intracellular lipids in oleaginous bacteria (Luo et al., 2019). Recently, wastewater sludge has emerged as a promising approach for promoting TAG biosynthesis by oleaginous microorganisms. Wastewater sludge provides a favorable environment enriched with cost-effective carbon and nutrient sources, facilitating the growth of these bacteria and the subsequent degradation of waste (Ambat et al., 2018). Furthermore, sewage sludge has garnered attention as a viable lipid source for biodiesel production, particularly through direct transesterification techniques using oleaginous bacteria (Mandolesi de Araújo et al., 2013) by harnessing the potential of wastewater sludge, oleaginous bacteria can efficiently convert the available carbon and nutrients into valuable lipids (Apriliana et al., 2025). The use of *Streptomyces* sp. in harnessing sludge for biodiesel production offers a sustainable and environmentally conscious approach to waste management and renewable fuel generation.

The increasing global sewage sludge production presents a significant environmental challenge, yet it also offers an opportunity for resource recovery and sustainable utilization. Among various valorization approaches, employing sludge as a substrate for oleaginous bacteria to produce bio-lipids represents a relatively novel and underexplored strategy. This study aims to investigate the potential of *Streptomyces* sp. for microbial lipid production from sewage sludge, followed by the conversion of these lipids into biofuels through transesterification. The use of *Streptomyces* sp. for bio-lipid production from sewage sludge is a novel approach, as no comprehensive studies have specifically addressed this combination. Several studies have demonstrated the lipid-producing potential of *Streptomyces* under specific conditions. *Streptomyces jeddahensis* has been shown to accumulate triacylglycerols under nitrogen-limiting conditions, using sodium octanoate, making it a potential candidate for biofuel production (Abdulkhair et al., 2025). Similarly, *Streptomyces coelicolor* STRP-19 has demonstrated the ability to degrade lignocellulosic biomass, such as rice husk, for lipid production (Lu et al., 2014). Furthermore, *Streptomyces* species have been used for organic waste degradation (Zhao and Wang, 2024; Zhang et al., 2025), but their application for lipid production from complex waste such as sewage sludge is scarce. Sewage sludge, a rich source of organic matter, primarily treated using microalgae (e.g., *Chlorella* sp.) (Song et al., 2024), oleaginous yeasts (e.g., *Yarrowia lipolytica*) (Moreno et al., 2024), and fungi (e.g., *Aspergillus oryzae*) (Garduño et al., 2024) due to their high lipid accumulation capacities. In the present study, the idea is to bridge the potential of *Streptomyces* for lipid accumulation and the use of high organic content sewage sludge for valorizing sewage sludge into valuable lipids for biodiesel production.

## 2 Materials and methods

### 2.1 Sampling of wastewater sludge

The sludge samples were collected from the clarifying tank of the sewage treatment plant situated in I-9, Islamabad, Pakistan (Latitude: 33° 39′36.5292″, Longitude: 73° 3′19.026). Subsequently, the samples were stored at 4°C for further utilization.

### 2.2 Physicochemical characterization of wastewater sludge

The sludge sample was initially characterized for color, temperature, odor, pH, Electrical Conductivity (EC), Total Dissolved Solids (TDS), COD, metal analysis by Inductive coupled plasma (ICP), Total Suspended Solids (TSS), water content, Nitrogen analysis, and carbon analysis.

### 2.3 Culture preparation

A pre-isolated oleaginous strain of *Streptomyces* sp. was obtained from NRRL, which is a collection of microorganisms and cultures maintained by the Agricultural Research Service (ARS) of the United States Department of Agriculture (USDA) located in Peoria, Illinois, USA. The strain was stored in glycerol at 4°C to maintain its viability. To initiate fresh cultures, 20 mL of LB media was prepared for the experimental culture, while 10 mL of LB media was prepared as control. Both cultures were then incubated at 30°C for 24 h. Initially, the purification process was carried out using the dilution plate technique on LB media. The bacteria were spread onto LB agar plates. These plates were then incubated at a temperature of 30°C for 24 h. After incubation, single colonies were carefully isolated from the LB agar plates.

#### 2.3.1 Characteristics of *Streptomyces* sp.

The *Streptomyces* sp. is characterized by gram staining and catalase test. Gram staining was performed using a clean slide. The smear turned purple, indicating that *Streptomyces* sp. is Gram-positive. For catalase testing, the media was prepared, and strains were grown on it. Further process was done after 24 h of incubation at 30°C After 24 h the glass slides were taken and using a sterilized inoculating loop, bacteria were placed on the slide in small amounts and 2–3 drops from 3% hydrogen peroxide were poured on the bacteria. There was no bubble formation which indicated catalase-negative activity in bacteria.

### 2.4 Experimental setup

The strain was tested for its lipid production potential using sludge. Various experiments were performed for optimization and sludge degradation Table 1. The incubation time of the bacterial culture was tested at different intervals, including 24 h, 48 h, 72 h, and 96 h, to determine the optimal duration for lipid accumulation. The pH levels of the growth medium were adjusted to values of 4–9 to evaluate their impact on lipid accumulation. Additionally, different temperatures ranging from 25°C to 40°C were investigated to determine the temperature range that promotes maximum lipid accumulation. The speed of the culture agitation, measured in revolutions per minute (RPM), was varied at 0, 150, and 250 to assess its influence on lipid accumulation. Furthermore, four different carbon sources, namely glucose, sucrose, dextrose, and starch, were tested to identify the carbon source that yields the highest lipid accumulation. Similarly, four nitrogen sources, including urea, yeast, ammonium chloride, and ammonium nitrate, were examined to determine the nitrogen source that optimally supports lipid accumulation. By optimizing these factors, the study aimed to identify the conditions that lead to the best results and maximum lipid accumulation in the strain. The experiments were conducted in triplicate (*n* = 3) to ensure reproducibility, and the results are reported as mean ± standard deviation (SD). Standard deviation was applied to assess the variability in the data, providing a measure of the consistency and precision of the experimental outcomes.

**Table 1:** Experimental conditions for optimization of lipid accumulation using sludge as substrate.

Experiments	Optimization levels					
Incubation (h)	24	48	72	96	–	–
Inoculation (%)	5	10	15	–	–	
pH	4	5	6	7	8	9
Temperature	25	30	35	40	–	–
Shaking condition (rpm)	0	150	250	–	–	–
Carbon source (0.5 g/L)	Glucose	Sucrose	Starch	Dextrose	–	–
Nitrogen source (0.05 g/L)	NH_4_Cl	Yeast	NH₄NO	Urea	–	–

### 2.5 Continuous shaking aerobic batch reactor (CSABR)

The lab-scale CSABR was developed to assess lipid accumulation under the optimized conditions. The CSABR is highly effective for conducting aerobic biological processes, particularly in waste treatment, biodegradation studies, and microbial cultivation. Furthermore, this system is simple to operate, as continuous shaking improves oxygen diffusion and bacterial mixing and ensures efficient nutrient availability to bacteria. In this experiment, four 250 mL batch containers were employed for the strain, waste, and control (the container was necessary due to shaking conditions). The experimental conditions for the bioreactor were adjusted at optimum conditions, including pH: 8.0, temperature: 30°C, and agitation: 150 rpm. During the operation, samples were collected over 4 days, during which measurements were taken for OD, CDW, and lipid accumulation. The strain demonstrated noteworthy potential for further investigation throughout all the optimization experiments and during the bioreactor study. Consequently, to gain a deeper understanding of its characteristics and potential applications, GC–MS and FTIR analyses were conducted on lipids extracted from *Streptomyces* sp.

### 2.6 Degradation kinetics

The pseudo-second-order model regards chemisorption as the rate-limiting mechanism of the process, in contrast to the pseudo-first-order model, which posits that physio-sorption restricts the rate at which the particles adsorb onto the adsorbent.

#### 2.6.1 Pseudo-first-order kinetic model

The pseudo-second-order model regards chemisorption as the rate-limiting mechanism of the process, in contrast to the pseudo-first-order model, which posits that phase adsorbent. When a reaction exhibits pseudo-first-order kinetics, the reaction may not be of the first order. This often occurs when one reactant’s concentration is much higher than the others, causing the concentration of the less prevalent reactant to become a determinant of the reaction rate (Revellame et al., 2020).

The model is represented by


$$\log\;(\mathrm{qe}-\mathrm{qt})=\log(\mathrm{qe})-\frac{k1}{2.303\;}\;t$$

Where k1 (min^−1^) is the first-order rate constant, and qt_1_ and qe_1_ (mg g − 1) represent the adsorption capacities at equilibrium and time t, respectively. The values of k1 and qe were determined from the slope and intercept of the linear plot of log (qe − qt) versus t.

#### 2.6.2 Pseudo-second -order kinetic model

The pseudo-second-order model regards chemisorption as the rate-limiting mechanism of the process, in contrast to the pseudo-first-order model, which posits that phase adsorbent. When a reaction exhibits second-order kinetics even if it is not a true second-order reaction, this is known as pseudo-second-order kinetics. This happens when the concentration of one reactant has a large impact on the reaction rate, giving the reaction a second-order appearance about that particular reactant (Oboh et al., 2013).


$$\frac{t}{\;\mathrm{qt}}=\frac{1}{k2qe^{2}}+(\frac{1}{\mathrm{qe}})\;t$$

Where K2 is the rate constant, the values of K2 and qe_2_ were determined from the slope and intercept of the linear plot of t/qt versus t.

The goodness of fit was assessed by comparing the coefficient of determination (R^2^) of both models. The model with the highest value and the closest predicted q_e_ to the experimental q_e_ was considered the best representation of the adsorption kinetics.

### 2.7 Analysis

The analysis of various parameters was performed using standard methods and protocols.

#### 2.7.1 Lipid content

Lipid content was calculated using the Bligh and Dryer method (Breil et al., 2017). The bacterial isolates were incubated for 96 h at a temperature of 30°C. After incubation, the cell biomass was obtained by centrifuging the culture at 10°C for 20 min at 10,000 rpm. This centrifugation step helps to separate the cells from the liquid medium. The cell pellets were washed twice with double-deionized water to remove any residual nutrients or media components. After washing, the cell pellets were dried at 50°C for 1 h. The tube with the dried cell pellets was then placed in an oven at 30°C for 24 h. This provides a quantitative measurement of the biomass obtained. In addition to CDW estimation, the lipid content in the cell biomass was obtained using the chloroform/methanol extraction method. The dried pellet biomass was mixed with a mixture of chloroform and methanol in a ratio of 2:1 (v/v). This mixture added 2.5 mL of chloroform and 1.25 mL of methanol. Additionally, 1.25 mL of 1 M NaCl solution was added to the same falcon tube. The lower layer containing the lipids was dried under a stream of N^2^ gas to obtain the dry lipid pellet. The lipid percentage was then calculated by dividing the weight of the lipids by the CDW.

#### 2.7.2 Fourier transform infrared spectroscopy

The lipids extracted from the optimized conditions in CSABR were used for FTIR analysis (Agilent technology, micro lab method, Range: 4000–400), to analyze the characteristic changes in surface functional groups (Lu et al., 2025). The sample was prepared, and the falcon tube lipid was shifted to another tube for drying purposes. The falcon tube was completely dried by nitrogen steam, which was further used for fatty acid analysis in FTIR, where different functional groups with wave number and density were obtained.

#### 2.7.3 GC–MS

The extracted lipids obtained from the strain were further analyzed using GC–MS (GCMS-5977B Agilent Technologies USA) to determine the type of lipid storage present. The operational parameters are provided in Table 2. To prepare the sample for analysis, the extracted lipids in the solvent were carefully transferred to separate vials using a syringe with a 0.2-micron size. A small amount of Na2SO4 (sodium sulfate) was added to remove any moisture content from the extracted sample. Na_2_SO_4_ acts as a drying agent. Finally, the prepared sample, free from solids and moisture, was transferred to 1.5 mL GC–MS vials. By subjecting the lipid extract to GC–MS analysis, it becomes possible to identify and characterize the specific lipid storage types present in the extracted sample.

**Table 2:** Operational conditions of GC–MS for lipid analysis.

Parameters	Condition
GC conditions	
Sample Inlet	GC
Injection source	GC ALS
Injection location	Front
Mass spectrometer	Enabled
Run time	36 min
Post run time	0 min
Setpoint	On
(Initial)	70°C
Hold time	3 min
Post run	50°C
Equilibration time	2 min
Max temperature	300°C
Slow fan	Disabled
Mode	Split
Heater	On 250°C
Pressure	On 8.8085 psi
Split ratio	20:01
Split flow	20 mL/min
MS conditions	
Jet clean	No cleaning
MS source	230 C maximum 250 C
MS Quad	150 C maximum 200 C

#### 2.7.4 Operational parameters

The COD of sludge samples was analyzed using the open reflex method (titration) described by (5220) the American Public Health Association (Association APH, 2005). The volatile solids of sludge samples were analyzed using the method described by the (5240) American Public Health Association. The fixed solids were analyzed using (5240) by the American Public Health Association. Nitrogen content was determined using the Kjeldahl method (4500) American Public Health Association. The estimation of total carbon was determined by ash percentage through the muffle furnace method (Pereira et al., 2006). Water content was analyzed by using (Sahadat et al., 2021). The reducing sugars were checked by the method of benedict testing (Simoni et al., 2002). The pH, EC, and TDS of the wastewater sample were taken by using a crimson multimeter (model number MM 40+). For metals analysis in the sludge, the samples were analyzed by ICP-OES.

#### 2.7.5 Transesterification of extracted lipids

The extracted lipids from CSABR were subjected to an acid-catalyzed process (Meher et al., 2006). A 10:1 ratio was employed, with 1 mL of methanol and 1 mL of chloroform added to 10 mL of lipid, along with 15% sulfuric acid. The mixture was placed in a hot bath at 100°C for 2.5 h. During the process, a distinct layer of biodiesel formed, which was carefully separated using a syringe.

### 2.8 Statistical analysis

In the present study, the experiments were performed with three replications to ensure reproducibility. The data set was analyzed using MS Excel software, and results were shown as mean with standard deviation ±. Standard deviation was applied to observe data variability and precision across replicates. Graphical representation includes standard deviation error bars to visually represent data dispersion.

## 3 Results

### 3.1 Physicochemical characteristics of wastewater sludge

The chemical properties of sludge hold immense significance for various applications. When considering sludge as a fertilizer or soil conditioner, assessing its fertilizer value becomes crucial. This study presents the concentrations of different heavy metals found in the sludge. The physical properties of the sludge encompass a range of features. Initially, the sludge emits an unpleasant odor. Its temperature measures at 25°C, while the color typically appears brownish. However, the sludge undergoes darkening and transformation over time, eventually becoming black. The reducing sugars in sludge samples were zero because no color change was observed, but the sample with 5% glucose showed a color change. Table 3 provides details on the various chemical properties of the sludge.

**Table 3:** Physico-chemical analysis of wastewater sludge.

Parameters	Values
pH	6.64 ± 0.01
EC	0.3 ± 0.03
TDS	815 ppm ± 0.33
COD	6,400 mg/L ± 0.01
TSS	300 mg/L ± 3.33
Total solids	3.5 mg/L ± 0.03
Total fixed solids	0.1 mg/L ± 0.01
Total Volatile solids	3.4 mg/L ± 0.03
Total Kjeldahl Nitrogen	0.19% ± 0.6
Total carbon	52.52% ± 0.33
Moisture content	96.5% ± 0.01
Metals concentration (mg/l)	
As	0.01
Cd	16.73
Co	0.20
Cr	0.01
Cu	0.72
K	21.21
Ni	0.01

### 3.2 Characteristic of *Streptomyces* sp.

The bacterial characterization, involving gram staining and a catalase test, indicates that the strains of *Streptomyces* sp. are gram-positive bacteria. Gram-positive bacteria, such as *Streptomyces* sp., accumulate more lipids possibly because they lack an outer membrane (Rajput et al., 2024), which allows for internal lipid storage and metabolic adaptability under stress conditions. In case of catalase activity, the results demonstrate the absence of catalase activity, as no bubble formation was observed during the test. Sewage sludge is a highly complex substance that subjects microorganisms to a variety of environmental stresses, such as variations in oxygen levels and nutritional availability. The catalase activity was measured to assess the bacterial stress response (Nivedita et al., 2024). Some bacteria regulate enzyme expression in response to nutritional conditions. The observed negative catalase activity in this strain may reflect a metabolic shift favoring lipid production over oxidative stress management, suggesting an adaptation strategy for lipid accumulation under sludge-derived stress conditions.

### 3.3 Optimization of reaction conditions for lipid

#### 3.3.1 Lipid accumulation

**Figure 1:**
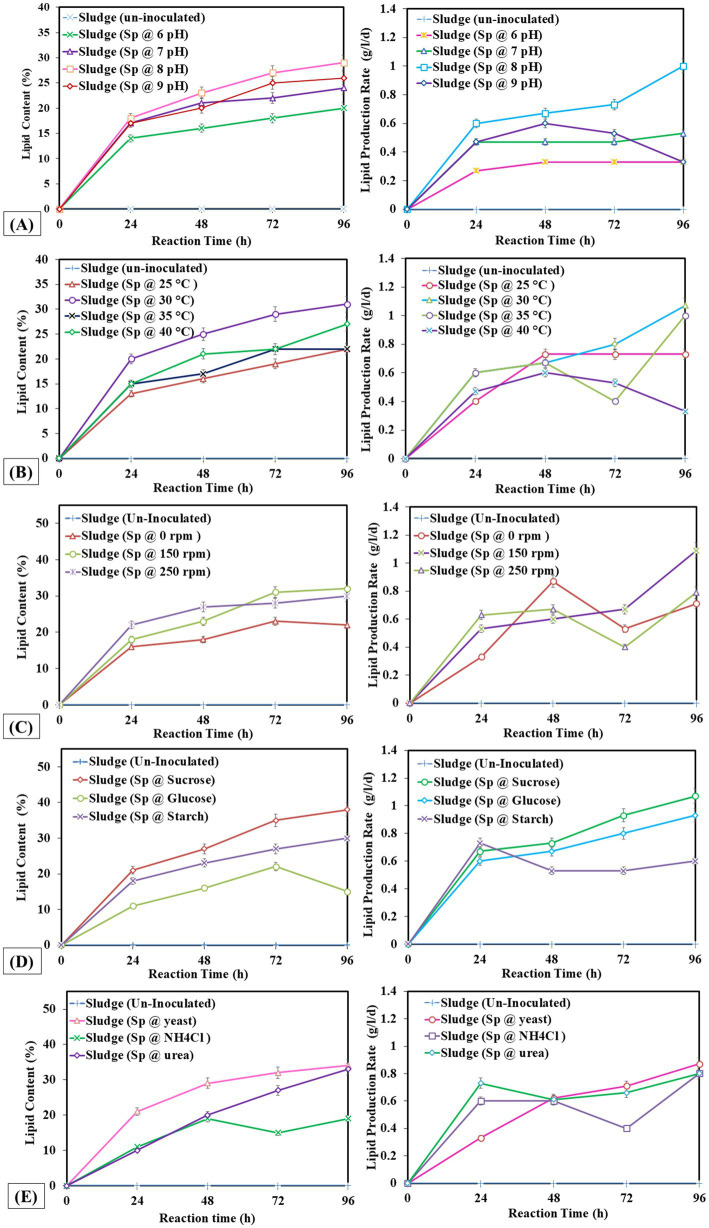
Lipid production rate and lipid percentage at different operating factors **(A)** pH, **(B)** temperature, **(C)** shaking, **(D)** carbon source, **(E)** nitrogen source.

Measuring lipid production rate (g/L/d) and content (%) is critical as the rate at which lipids are produced is an essential indicator of how well lipid synthesis mechanisms work throughout a given period. Second, knowing the lipid content helps estimate the total lipid output and quality since it provides information about the concentration of lipids in the biomass or culture media. This measurement helps determine the ideal conditions that maximize the accumulation of lipids by comparing various environmental parameters. The study found that extended incubation of 96 h and 5% v/v inoculum size was ideal. At the same time, pH 8 and 30°C were optimal for enhanced lipid production rates, and agitation at 150 rpm improved lipid synthesis efficiency. In Figure 1A, the highest lipid production was observed at pH 8, with a lipid yield of 3 g/L, a lipid production rate of 1 g/L/d, and a lipid percentage of 29%. In Figure 1B, the highest lipid production was observed at 30°C with a lipid yield of 3.13 g/L, a production rate of 1.07 g/L/d, and a lipid percentage of 31%. Figure 1C, the highest lipid production was observed at 150 rpm with a lipid percentage of 32%. In Figures 1D,E, the highest lipid production (38 and 34%) was observed with sucrose (carbon source) and yeast (nitrogen source) respectively. Therefore, sucrose was the best carbon source, and yeast proved effective for nitrogen supply to achieve higher lipid production.

#### 3.3.2 Cell dry weight

**Figure 2:**
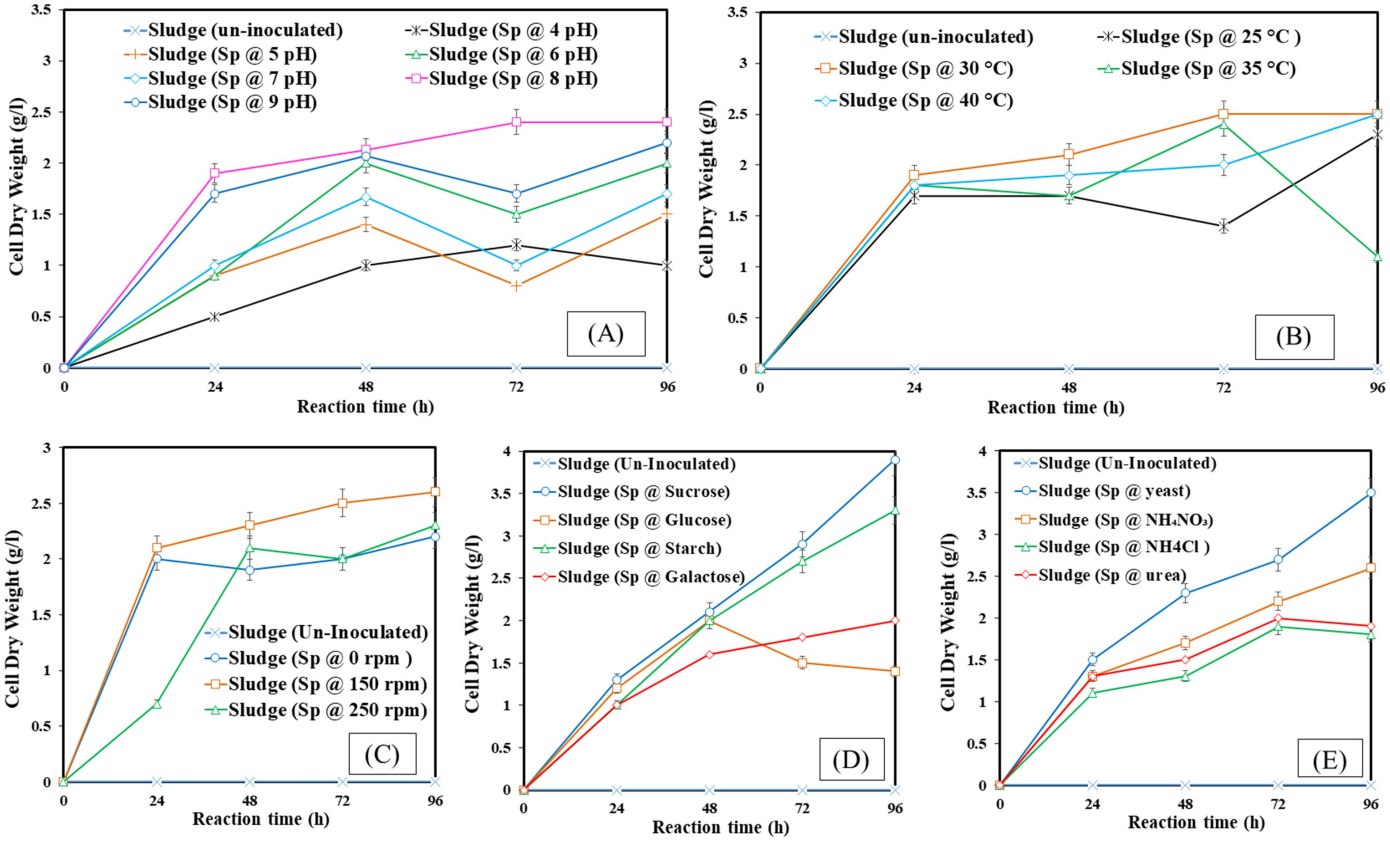
Cell dry weight at different operating factors **(A)** pH, **(B)** temperature, **(C)** shaking, **(D)** carbon source, **(E)** nitrogen source.

CDW represents the total biomass after all liquid components have been eliminated and is the metric used to quantify the amount of dried biomass generated. Calculating the CDW is crucial because it offers a consistent way to measure biomass production, making it possible to evaluate the development and output of microorganisms. The experimental conditions: pH 8, 30°C temperature, 150 rpm agitation, sucrose as a carbon source, and yeast as a nitrogen source were identified as optimal. Figure 2A illustrates that the optimal pH for dry biomass production is pH 8, with a production yield of 2.4 g/L. In Figure 2B, the optimal temperature for dry biomass production is 30°C, with a production yield of 2.5 g/L. In Figure 2C, the optimal shaking condition for dry biomass production is 150 rpm, with a production yield of 2.6 g/L. In Figure 2D, sucrose is the optimal carbon source for dry biomass production, with a production yield of 3.9 g/L. Figure 2E shows that yeast is the optimal nitrogen source for dry biomass production, with a production yield of 4.0 g/L. Conditions that are not ideal might limit cell development and biomass formation, which lowers CDW values. These conditions include acidic pH, lower temperatures, insufficient agitation, and unavailability of vital nutrients.

#### 3.3.3 Chemical oxygen demand

**Figure 3:**
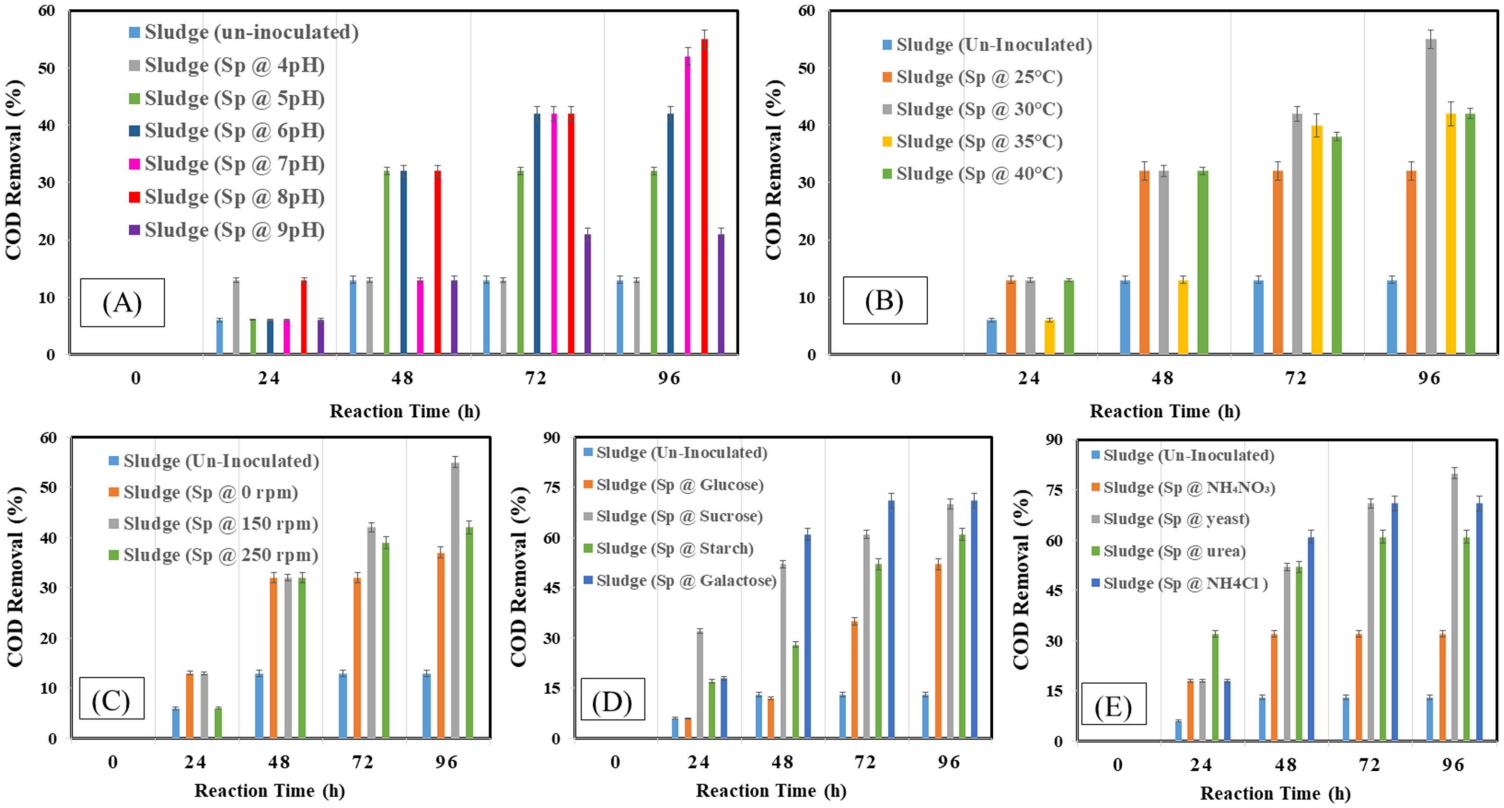
Chemical oxygen demand at different operating factors **(A)** pH, **(B)** temperature, **(C)** shaking, **(D)** carbon source, **(E)** nitrogen source.

The effect on COD removal as indicator of organic matter constituents during the reaction time of 96 h is presented in Figures 3A–E. The capacity of bacteria to degrade waste was at its peak, with a 5% (v/v) resulting in a COD removal of 52%. It was discovered that pH 8 was ideal for removing 55% organic compound. The bacteria attained a comparable clearance rate of 55% COD at 30°C. With a 55% removal efficiency, Waste degradation was maximized by agitation at 150 rpm with 80% removal rates; sucrose and yeast were shown to be the most successful in encouraging COD elimination. Glucose and ammonium chloride, on the other hand, produced lower clearance rates, highlighting their less advantageous effects on waste breakdown. These results emphasize the ideal circumstances to maximize the effectiveness of bacteria’s elimination of COD.

#### 3.3.4 Organic matter degradation efficiency

**Figure 4:**
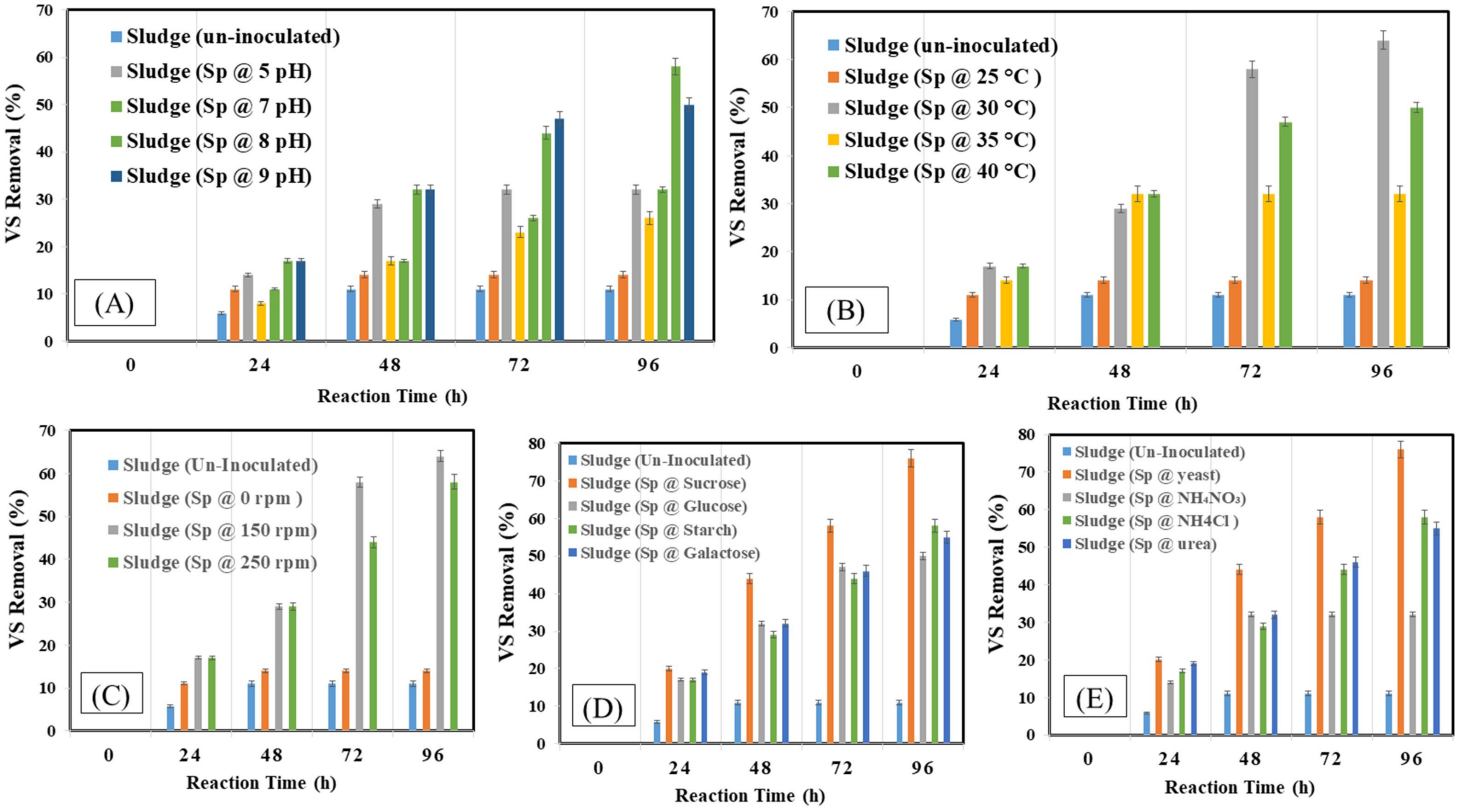
Volatile solids at different optimization factors **(A)** pH, **(B)** temperature, **(C)** shaking condition, **(D)** carbon source, **(E)** nitrogen Source.

The organic matter removal efficiency was measured as percentage-VS removal which is presented in Figures 4A–E under different operating conditions. The study found that 96 h was the ideal incubation period for VS elimination, with a 38% reduction. Examining the effect of inoculum size, it was found that a 5% inoculum size produced the highest VS removal 38% when the proper balance of bacteria was present to break down the solids and a 15% inoculum size produced the lowest removal of 14%. The removal efficiency peaked at pH 8 at 58% and decreased to 14% at pH 4. Comparably, a temperature of 30°C produced a clearance rate of 64% since it coincided with the bacteria’s ideal growth temperature, as opposed to the 14% at 25°C. While no agitation at 0 rpm produced a removal rate of 14%, agitation at 150 rpm maximized VS removal to 64%. The most efficient carbon source was sucrose, which had a 76% removal rate as opposed to glucose’s 50% removal rate. With a 76% clearance rate, yeast was shown to be the most effective nitrogen source.

#### 3.3.5 Total dissolved solids

**Figure 5:**
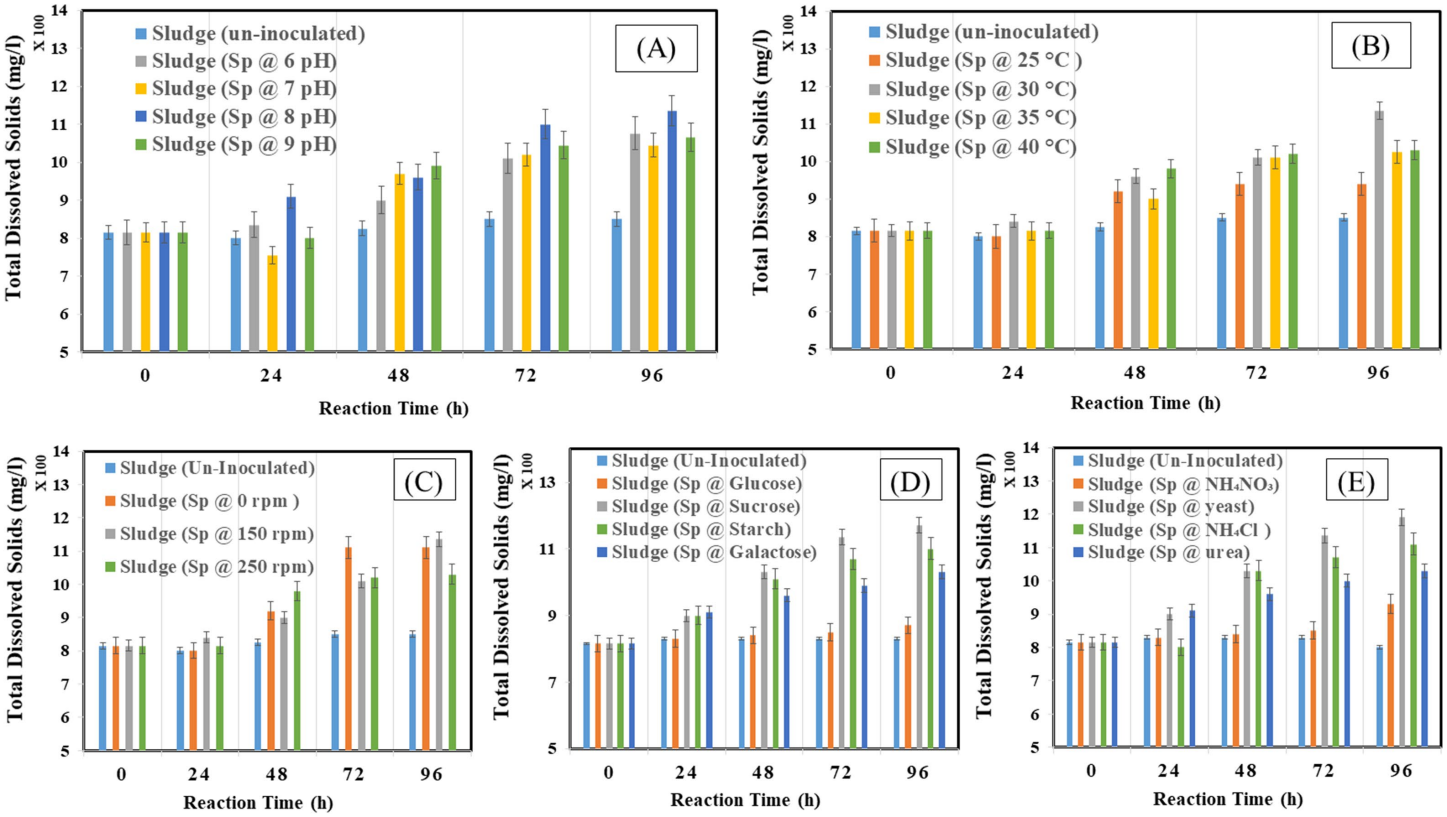
Total dissolved solids at different optimization factors **(A)** pH, **(B)** temperature, **(C)** shaking condition, **(D)** carbon source, **(E)** nitrogen source.

The effect on TDS under different operating conditions is illustrated in Figures 5A–E. It was observed that the TDS increased at 48 and 72 h with 900 mg/L and 950 mg/L, and decreased at 96 h with the value of 850 mg/L. The results show a continuous rise in TDS by bacterial inoculation. The increase is more obvious at 96 h for 5%v/v with 950 mg/L. the samples inoculated with 10% v/v and 15%v/v show constant value from 48 to 96 h 910 mg/L. In Figure 5A, the results represented a continuous rise in waste at pH 8 with 1,135 mg/L at 96 h and lowest at pH 4 with 980 mg/L. In Figure 5B, the results represented a continuous rise in waste at a temperature of 30°C with 1,135 mg/L at 96 h and lowest at 25°C with 940 mg/L. In Figure 5C, the highest TDS was observed at 150 rpm with 1,135 mg/L at 96 h and lowest at 0 rpm with 1,005 mg/L. In Figure 5D. The results represented the highest TDS at the sucrose source, with 1,170 mg/L at 96 h, and the lowest at the glucose source, with 840 mg/L. In Figure 5E the highest TDS was observed at the yeast source with 1,190 mg/L at 96 h and lowest at the ammonium nitrate source with 900 mg/L.

### 3.4 Degradation kinetics

**Figure 6:**
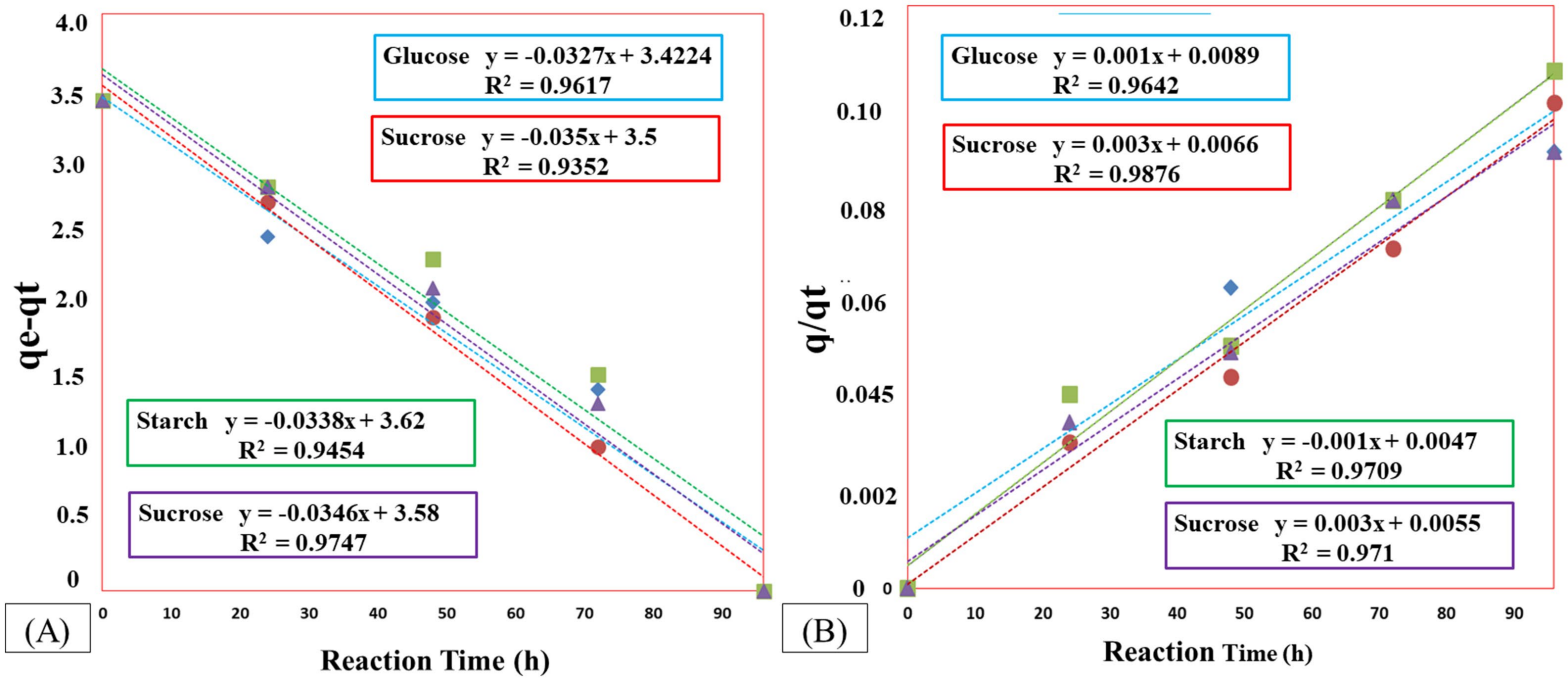
COD degradation kinetics using different carbon sources under optimized conditions **(A)** Pseudo-first-order kinetics – the x-axis represents time (h), and the y-axis shows the COD concentration. **(B)** Pseudo-second-order kinetics – the x-axis shows time (h), and the y-axis represents q/qt. In both graphs, data points indicate experimental values, while the fitted lines represent the respective kinetic models applied.

The rate constants for COD degradation kinetics in pseudo-first-order and pseudo-second-order reactions are presented in Figures 6A,B. The calculated adsorption capacities (qe1 and qe2) for pseudo-first-order and pseudo-second-order reactions, respectively, are expressed in milliliters per liter (ml/L). The values of the rate constants and the coefficient of determination (R^2^) are provided in Table 4. The R^2^ values, close to unity for pseudo-first order, indicate well-fitting results for COD degradation using the pseudo-first-order kinetic model. The highest values for rate constant k1 were observed with sucrose, respectively.

**Table 4:** The value of a kinetic parameter for COD degradation in source-optimized.

Operational parameters	Pseudo first-order model			Pseudo second order model		
Carbon source	q_e1_ (ml/L)	k_1_ (min^−1^)	R^2^	q_e2_ (ml/L)	k_2_ (min^−1^)	R^2^
Glucose	985.75	−0.032	0.961	985.75	0.001	0.964
Sucrose	1,293	−0.035	0.995	1,293	0.003	0.987
Starch	985.75	−0.033	0.945	985.75	0.001	0.97
Dextrose	1139.25	−0.034	0.974	1139.25	0.003	0.971

### 3.5 Performance in continuous shaking aerobic batch reactor (CSABR)

**Figure 7:**
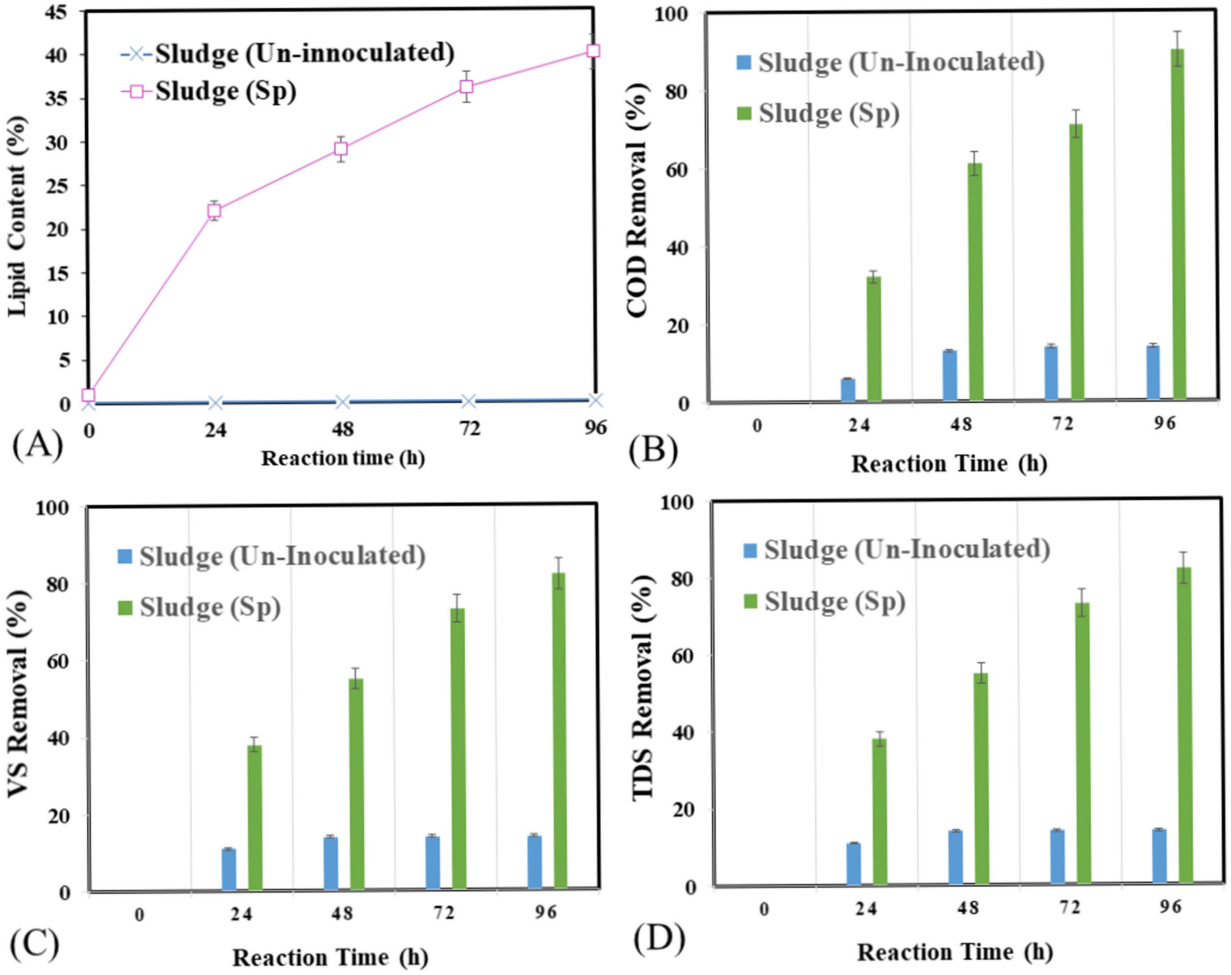
Lipid accumulation and degradation potential of *Streptomyces* sp. under optimized condition in bioreactor **(A)** lipid accumulation in g/l, **(B)** COD removal %, **(C)** VS removal %, **(D)** TDS.

A continuous shaking aerobic batch bioreactor was used to observe the effect of optimized conditions on lipid production by *Streptomyces* sp. at the pilot scale. The operation was conducted at pH 8 with 5% v/v inoculation, 30°C temperature, and 150 rpm shaking (Figures 7A–D). For nutrients, sucrose (carbon source) and yeast (nitrogen sources) were added for 96 h. The lipid production was observed with a lipid yield of 4.80 g/L, a lipid production rate of 1.80 g/L/d, and a lipid percentage of 40%. No lipid production was observed in control samples. In Figure 7B COD removal at optimized condition was 90% at 614 mg/ and a control sample with 13% at 5529 mg/L. In Figure 7C, the highest VS removal percentage occurred at 96 h 82% at 0.6 mg/L, and in the control sample, 14% was used with 3 mg/L. In Figure 7D shows the impact of the optimized conditions on TDS from 24 to 96 h. At 24 h980 mg/l than at 48 h 1,015 mg/L than at 72 h 1,120 and at 96 h 1,200 mg/L. Overall, the control sample had an increasing trend, and the TDS was also increased to 900 mg/L. the variation in pH at 24 h was 7.9 at 48 h the pH observed was 7.2 and at 72 h the pH was 7.5 and at 96 h pH increased to 8. In the control sample, the pH almost showed no variation. The impact of the optimized condition on EC variations from 24 to 96 h. At 24 h, EC is 0.6. At 48 h, 0.8. At 72 h, 0.9; and at 96 h, 1.1 overall, showing an increasing trend, and in the control sample, EC shows no variations.

#### 3.5.1 Lipid characterization

##### 3.5.1.1 FTIR

**Figure 8:**
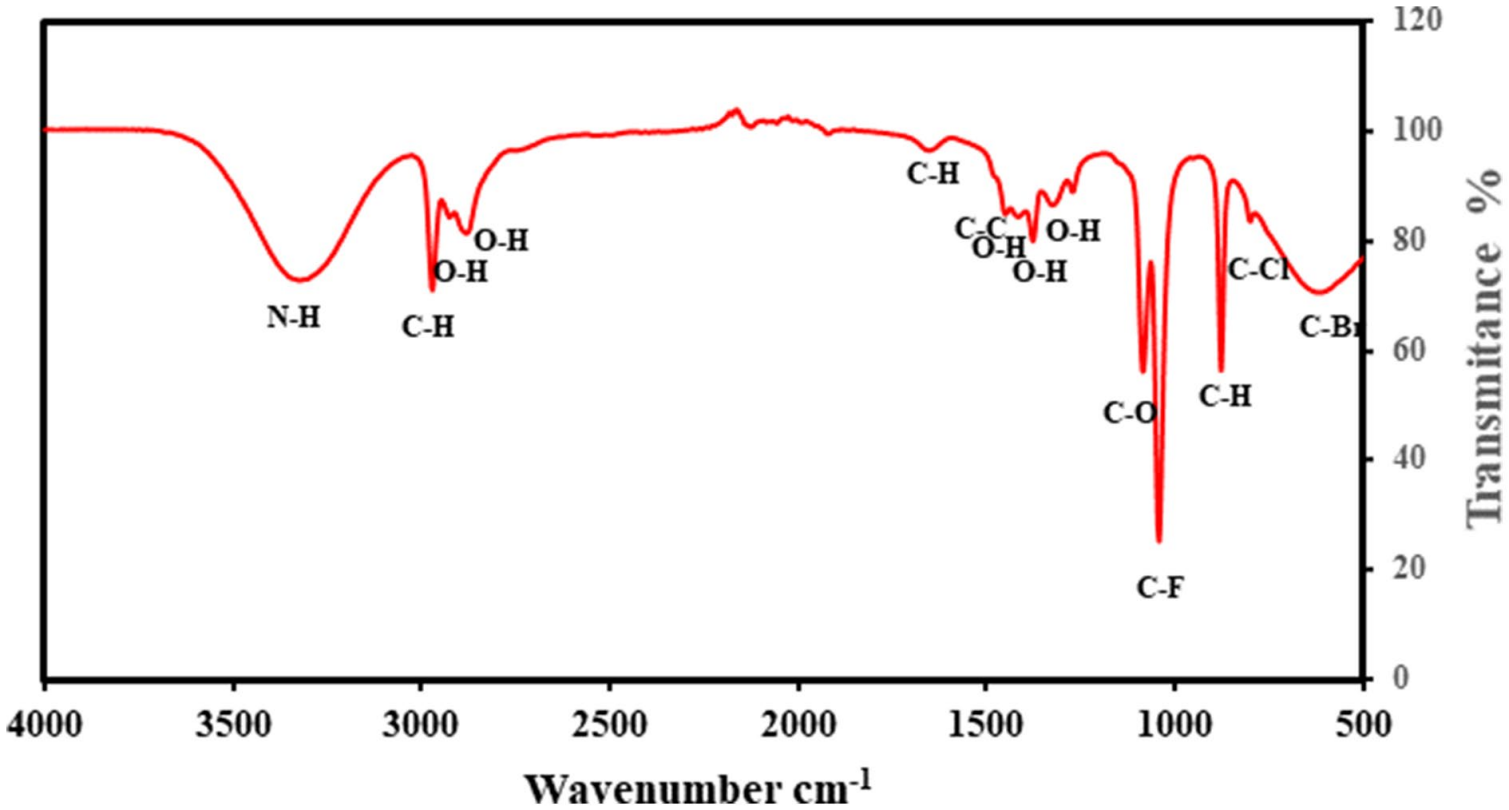
Fourier-transform infrared (FTIR) spectrum analysis of lipid extracted from *Streptomyces* sp. cultured in bioreactor sample. The x-axis represents wavenumber (cm^−1^), and the y-axis represents transmittance (%).

The identification of functional groups in the lipids was analyzed using FTIR, as illustrated in Figure 8. The magnitude of peaks at various wavenumbers indicates the presence of specific functional groups characteristic of *Streptomyces* sp. At 3321 cm^−1^, the N–H stretching vibration confirms the presence of aliphatic primary amines, while the peaks at 2974 cm^−1^, 2,925 cm^−1^, and 2,881 cm^−1^ correspond to C–H and O–H stretching vibrations, signifying the presence of alkanes and alcohols. The absorption bands at 1654 cm^−1^ (C–H bending) indicate aromatic compounds, whereas the peaks at 1453 cm^−1^ and 1,416 cm^−1^ correspond to C–C (in-ring) stretching and O–H bending, associated with alkanes and carboxylic acids, respectively. Further, the peaks at 1,379 cm^−1^ and 1,326 cm^−1^ represent O–H stretching, confirming the presence of phenols. The detection of aliphatic ethers (1,088 cm^−1^). These findings suggest the presence of biofuel-related compounds, including hydrocarbons and oxygenated functional groups, which play a crucial role in biodiesel synthesis. Overall, the FTIR verified the presence of important functional groups linked with biofuel production such as carboxyl, hydroxyl, and alkane groups, which are present in fatty acids and their derivative compounds.

##### 3.5.1.2 Lipid profile (GC–MS)

**Figure 9:**
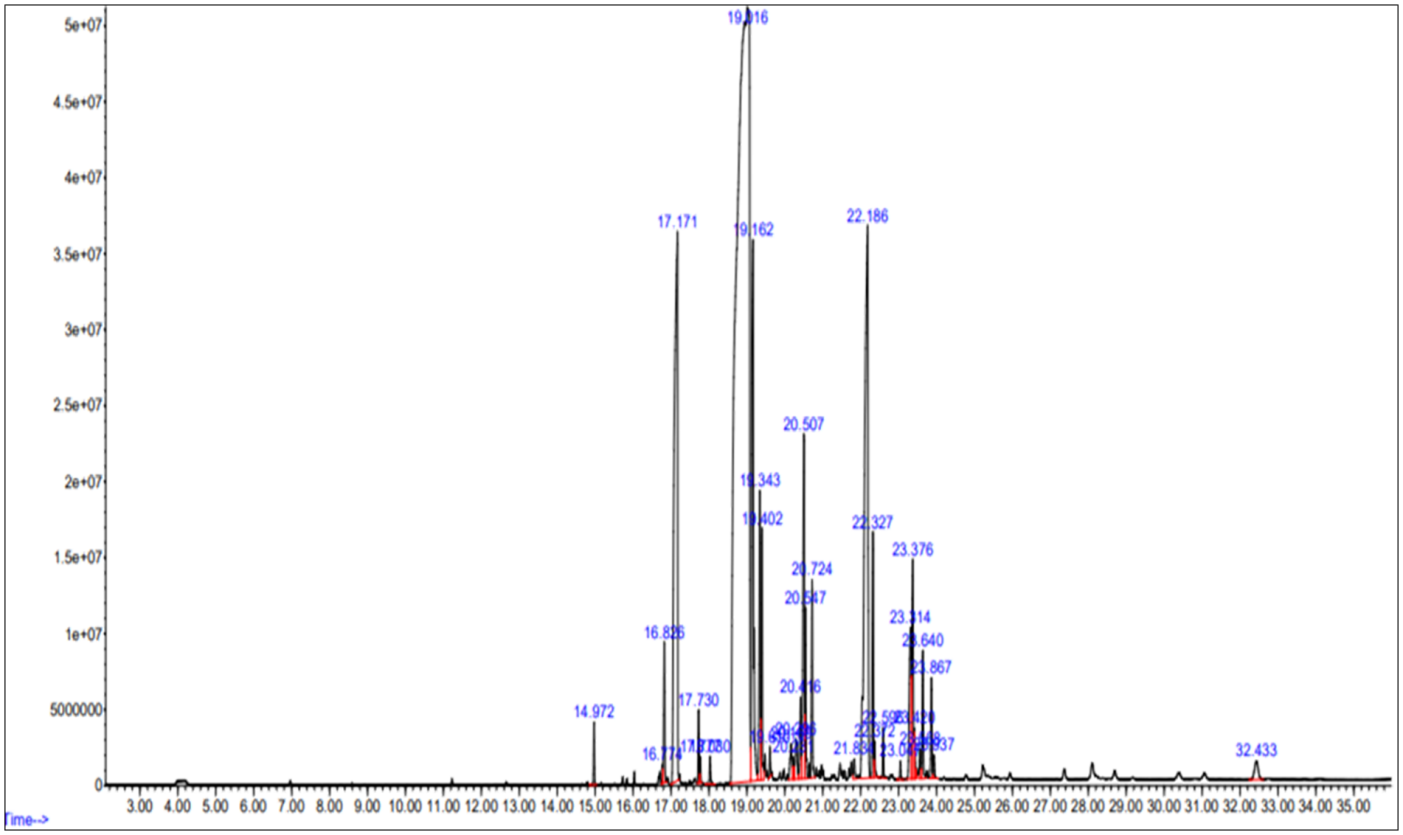
Gas chromatography–mass spectrometry (GC–MS) analysis of fatty acid methyl esters (FAME) derived from *Streptomyces* sp. cultured in bioreactor. The x-axis represents retention time (min), and the y-axis represents relative abundance (%).

The lipids extracted from *Streptomyces* sp. cells underwent GC–MS analysis, revealing the presence of various hydrocarbon compounds along with fatty acids (Figure 9). The analysis identified several free fatty acids (FFA), including heptadecanoic acid (margaric acid), hexadecanoic acid (palmitic acid), and 9-octadecenoic acid (oleic acid), which were among the most predominant compounds. Notably, the presence of hexadecanoic acid methyl ester (C₁₇H₃₄O₂) 25.11% and 9-octadecenoic acid methyl ester (C₁₉H₃₆O₂) 8.68% indicates a significant potential for biofuel applications due to their known roles in biodiesel production. The detailed fatty acid profile of the extracted lipids from bacterial cells grown on LB media is presented in Table 5.

**Table 5:** Fatty acid profile of the extracted lipids from the bacterial cells grown on LB media for determination of the potential of *Streptomyces* sp. to store lipids in the cell biomass (Temp 30°C; pH 8).

Peak	R.T	Area%	Compound	Compound category
3	8.560	2.46	Heptadecanoic acid	FFA
7	17.012	25.11	Hexadecanoic acid, methyl ester	FFA
8	17.334	2.41	n-Hexadecanoic acid	FFA
10	18.643	8.68	9-12-Octadecenoic acid, methyl ester 9-Octadecenoic acid, methyl ester 8-Octadecenoic acid, methyl ester	FFA

FFA, Free fatty acids.

#### 3.5.2 Production of bio-diesel from extracted lipids

The transesterification was conducted using acid catalysis methods. The biodiesel underwent testing for its properties, allowing for comparing all the compounds with diesel. The obtained biodiesel weighed 1 mL from 10 mL of lipid. Figure 9 illustrates the GC–MS analysis, revealing the FAME (Fatty Acid Methyl Ester) profile of the composition of various fatty acids present in transesterified biodiesel from *Streptomyces* sp., as tabulated in Table 6. Saturated fatty acids constitute 46.97% of the total, with specific breakdowns including 29.37% hexadecanoic acid (palmitic acid), 5.15% octadecenoic acid (stearic acid), 0.63% tetracosanoic acid (lignoceric acid), and 1.47% docosanoic acid (behenic acid). Unsaturated fatty acids are more predominant, accounting for 52.79% of the total composition. This includes 14.45% 9-octadecanoic acid (oleic acid), 5.70% 9–12-octadecanoic acid (linoleic acid), and 4.18% heptadecanoic acid (margaric acid). Other unsaturated fatty acids in the profile are 1.15% arachidic acid, 7.53% 13-docosenoic acid (erucic acid), 1.38% methyl oleate, and 0.78% methyl stearate.

**Table 6:** FAME of biodiesel from *Streptomyces* sp.

Peak no.	Common name	Fatty acid chain	CAS	Fatty acid conc.(%of total FAME)
12	Stearic acid (SFA) Octadecanoic acid, methyl ester	(C18:0)	000111-61-5	5.15
5	Palmitic acid (SFA) Hexadecanoic acid, methyl ester	(C16:0)	000112-39-0	29.37
27	Lignoceric acid (SFA) Tetracosanoic acid, methyl ester	(C25:0)	002442-49-1	0.63
19	Behenic acid (SFA) Docosanoic acid, methyl ester	(C22:0)	000929-77-1	1.47
4	Pentadecanoic acid, methyl esters (SFA)	(C15:0)	005129-60-2	10.04
8	Oleic acid (MUFA) 9-octadecanoic acid, methyl ester (E)-	(C18:1)	001937-62-8	14.45
22	Linoleic acid (PUFA) 9-12-octadecanoic acid, methyl ester (Z,Z)	(C18:2)	002277-28-3	5.70
16	Arachidic acid (PUFA) Eicosanoic acid methyl ester	(C20:1)	001120-28-1	1.15
21	Tricosanoic acid, methyl esters	(C23:0)	002433-97-8	0.09
18	Erucic acid (MUFA) Cis-docosenoic acid, methyl ester	(C22:1)	001120-34-9	7.53
11	Palmitolic acid (MUFA) 9 hexadecanoic acid methyl ester	(C16:1)	001120-25-8	0.91
7	Heptadecanoic acid mehyl ester (MUFA)	(C17:1)	001731-92-6	4.18

SFA, Saturated fatty acids; MUFA, Mono unsaturated fatty acids; PUFA, Poly unsaturated fatty acids.

## 4 Discussion

According to various studies, heavy metals such as Cd, Cu, Cr, Ni, Pb, Zn, Mn, Co, Hg, As, Fe, Na, K, Ca, Mg, CN, and B are present in sewage sludge. The presence of Cd suggests waste originates from fertilizer-using agricultural areas and industries like plating, plastic production, or battery manufacturing (Sarma et al., 2019). The highest levels are for Zn and Cu, followed by Cr, Ni, Pb, and Cd. Interestingly, there is no variation in total metal concentration between municipal and industrial wastewater treatment plants (Iloms et al., 2020). Furthermore, Badza et al. (2021) reported TOC at 30.31% and TKN at 6.40%, used as indicators of contamination. The carbon level of untreated samples (30–40%) is linked to the sludge’s origin. COD measures oxygen consumption by organic matter. The complex class of actinomycetes, particularly *Streptomyces* sp., is crucial to natural and artificial habitats. This study investigates the growth rate and characteristics of *Streptomyces* sp. *KB1,* including aerial and substrate mycelium. It displays gram-positive traits and produces favorable outcomes in catalase testing (Chawawisit et al., 2015). Maintaining optimal temperature conditions enhances bioactive compound production, as supported by studies such as Al-Dhabi et al. (2016), which observed similar preferences for *Streptomyces species* on fruit waste. Optimizing inoculum size is critical for bioactive compound (BC) production. BC yield decreased with inoculum concentrations lower or higher than 5% (v/v), as excessive inoculum led to resource competition, aligning with the findings of Zhang et al. (2014). *Streptomyces* sp. thrives in neutral to alkaline pH (7–11), with optimal growth at 7–8, while enzymes perform best at pH 10 (Yun et al., 2018). Temperature is key in lipid formation, affecting triacylglycerol generation. Lipase activity peaks between 30 and 50°C, with the highest production at 30°C, aligning with the findings of Suresh et al. (2021) on *Streptomyces species* BC production. Shaking at 150–250 rpm ensures proper aeration and prevents bacterial settling (Rakesh et al., 2014). The availability of carbon and nitrogen impacts triglyceride synthesis. High glucose but low nitrogen stimulates triglyceride production. In a study, Sangkharak et al. (2020) found fruit waste yielded 20.3 g/L FFA with sucrose as the carbon source. The yeast produced 17.05 g/L biomass, with ammonium chloride favoring lipid presence, aligning with Tr et al. (2012). Lipid accumulation in *Streptomyces* sp. decreased with NaNO_3_ addition, highlighting sensitivity to the nutrient environment. FTIR results align with the findings of Ambarwati et al. (2020) who reported similar functional groups in microbial lipid-based biodiesel. The key functional groups: N–H stretching (amines), C–H (alkanes) O–H stretching (alcohols), and C=O stretching (carbonyl) compounds, are important in lipid based biofuels (Hoekman et al., 2011). These functional groups correspond palmitic acid (hexadecanoic acid), oleic acid (9-octadecenoic acid), and their methyl esters detected in GC–MS spectra which are normally used in biodiesel production. The absorption band at 1654 cm^−1^ corresponds to C-H bending of aromatic compounds, while the strong band at 1,088 cm^−1^ is associated with C-O stretching in aliphatic ethers, both of which are important for fuel stability (Maleki-Kakelar and Mashinchian, 2024). The presence of carboxyl (-COOH) bending at 1416 cm^−1^ and phenolic O-H stretching at 1326 cm^−1^ suggests that the extracted lipids contain the necessary functional groups for biodiesel synthesis (Karmakar et al., 2018). These carboxylic acid (-COOH) functional groups detected in FTIR spectra arrange in a line with the FFA detected in GC–MS, attributed to the extracted lipids which are essential precursors for biodiesel synthesis. Hexadecanoic acid (palmitic acid) was reported by Mothana et al. (2022) resulting from the conversion of esters from *Streptomyces 1S1* oil to biodiesel with heptadecanoic acid (margaric acid) and octadecanoic acid (stearic acid) also found in *Streptomyces* species (El-Namoury et al., 2020). The presence of these long-chain fatty acids supports the biofuel potential of *Streptomyces* sp., aligning with previous studies on microbial biodiesel production. The predominance of C17, C19, and C20 methyl esters in the analysis further confirms the suitability of *Streptomyces* sp. as a renewable biodiesel feedstock (Jafarihaghighi et al., 2020). A higher concentration of palmitic acid in the blended biodiesel fuel enhances oxidative stability and increases the cetane value. Meanwhile, the presence of oleic acid influences ignition quality, combustion heat, and lubricity of the biofuel (Verma et al., 2024).

## 5 Conclusion

This work demonstrates the enormous potential of pre-isolated bacterial strains, *Streptomyces* sp., for lipid synthesis employing sludge as a substrate. The results show that this strategy provides a combined benefit of biolipid-based fuel production with effective waste management. Further confirming the ability of lipids recovered from these bacterial strains to produce biofuel is the identification of fatty acids in the lipid profile using GC–MS analysis, which opens the door for potential scalability from lab to industrial levels. Trials show how cost-effective waste can be successfully used with little additional carbon needed, leading to considerable lipid buildup and noticeable decreases in pollution loads like VS, COD, and TDS elimination leading to considerable lipid buildup and noticeable reductions in pollution loads such as VS, COD, and TDS. This research is, therefore, of utmost importance for developing biolipid-based fuel generation using *Streptomyces* sp. and serves as a reliable resource for long-term waste minimization strategies. Biodiesel from the oleaginous bacteria provides environmental benefits as it reduces greenhouse gas emissions and contributes to waste management. To improve process efficiency and commercial feasibility, future research should focus on scaling up the system, investigating genetic modifications of Streptomyces, and adopting advanced fermentation methods. Furthermore, implementing this bio-lipid synthesis strategy into wastewater treatment systems may provide a dual advantage of waste valorization and bioenergy generation, necessitating pilot-scale investigations to determine its practical applicability.

## Data Availability

The original contributions presented in the study are included in the article/supplementary material, further inquiries can be directed to the corresponding author.
